# Inflammatory Foot Involvement in Spondyloarthritis: From Tarsitis to Ankylosing Tarsitis

**DOI:** 10.3389/fmed.2021.730273

**Published:** 2021-10-08

**Authors:** José Pablo Romero-López, Dirk Elewaut, César Pacheco-Tena, Rubén Burgos-Vargas

**Affiliations:** ^1^Laboratorio A4, Carrera de Médico Cirujano, Facultad de Estudios Superiores Iztacala, Universidad Nacional Autónoma de México, Tlalnepantla de Baz, Mexico; ^2^Laboratorio de Inmunología Clínica 1, Posgrado en Ciencias Quimicobiológicas, Escuela Nacional de Ciencias Biológicas, Instituto Politécnico Nacional de México, Ciudad de México, Mexico; ^3^Ghent University Hospital, Ghent University, Ghent, Belgium; ^4^Facultad de Medicina, Universidad Autónoma de Chihuahua, Chihuahua, Mexico; ^5^Department of Rheumatology, Hospital General de México, “Dr. Eduardo Liceaga”, Ciudad de México, Mexico

**Keywords:** spondyloarthritis, ankylosing tarsitis, juvenile onset spondyloarthritis, foot arthritis, mechanical stress

## Abstract

Spondyloarthritis (SpA) is a group that includes a wide spectrum of clinically similar diseases manifested by oligoarticular arthritis and axial or peripheral ankylosis. Although axial SpA is predominant in Caucasians and adult-onset patients, juvenile-onset and Latin American patients are characterized by severe peripheral arthritis and particularly foot involvement. The peripheral involvement of SpA can vary from tarsal arthritis to the most severe form named ankylosing tarsitis (AT). Although the cause and etiopathogenesis of axSpA are often studied, the specific characteristics of pSpA are unknown. Several animal models of SpA develop initial tarsitis and foot ankylosis as the main signs, emphasizing the role of foot inflammation in the overall SpA spectrum. In this review, we attempt to highlight the clinical characteristics of foot involvement in SpA and update the knowledge regarding its pathogenesis, focusing on animal models and the role of mechanical forces in inflammation.

## Introduction

Spondyloarthritis (SpA) is a group of chronic inflammatory diseases of the entheses and the synovial membrane of the joints, tendons, and bursae that affects the spine, the sacroiliac joint, and peripheral sites (1, 2). Currently, SpA is known as axial SpA (axSpA) (3, 4) or peripheral (pSpA) (5). Ankylosing spondylitis (AS) represents the most severe form of SpA in which episodes of disease activity merge with chronic irreversible manifestations such as bone proliferation and ankylosis. According to ASAS classification, the “SpA” name is kept, and ankylosing spondylitis (AS) corresponds to radiographic axSpA (r-axSpA). While axSpA predominates at onset and through disease's course in European and European descendants (6), the combination of axSpA with pSpA is the clinical pattern most frequently found in Latin America (7–16), India (17, 18), the Middle East (19), and Asia (20).

In the past, studies on SpA referred to peripheral involvement in children, adolescents, and young adults (21, 22). Usually, peripheral involvement was described as an asymmetrical affection of the lower limbs. Regarding adult-onset disease, particularly AS, the peripheral disease became recognized in the mid-1970s (23, 24) as part of AS and disorders such as reactive arthritis (ReA) (formerly Reiter's syndrome), psoriatic arthritis (PsA), Crohn's disease, ulcerative colitis, and undifferentiated SpA.

Amor et al. (25) first included peripheral arthritis of the lower limbs among the SpA criteria. Then, the European Spondyloarthropathy Study Group (ESSG) proposed a classification system with two entry arms axial and peripheral (26). This idea gained recognition in the ASAS classification as pSpA. In the meantime, mid-foot arthritis, enthesitis, or tarsitis appeared as important manifestations in adolescents or young adults with AS (27).

It was challenging to assess enthesopathy even though the concept of “the entheseal organ” turned fundamental in understanding the disease's pathophysiology (28–30). Synovitis and particularly enthesitis, have been the target for studying cellular infiltrates, pro-inflammatory cytokines, and bone proliferation. As discussed below, the mechanisms leading to such phenomena include mechanic forces that act upon mechano-receptors, HLA-B27, ERAP1, and IL-23. The approach to studying the disease's pathogenesis has been driven from two paths: throughout animal models and human surgical samples.

## Foot Involvement and Tarsitis

Mid-foot arthritis, also known as, tarsitis, is a prominent feature in adolescents and young adult males with SpA (31, 32). Most adolescents and young adults have recurrent lower-limb arthritis and enthesitis combined with <20% axial symptoms. Five to 10 years later, 75% of such patients fulfill the AS criteria (33, 34). In contrast to juvenile-onset SpA (JoSpA), adults have back pain and 5 to 10 years later inflammatory back pain alongside sacroiliitis on magnetic resonance imaging (MRI) and radiographic studies (5, 35–39). Identifying the characteristic involvement of peripheral arthritis and enthesitis and its differentiation from other forms of juvenile idiopathic arthritis (JIA) as early as possible allows the use of biologic disease-modifying anti-rheumatic drugs (bDMARDs) years before the appearance of the spinal and sacroiliac joints symptoms (21). The same applies to eligibility criteria and outcome measures in clinical trials on the efficacy and safety of bDMARDs. Besides peripheral disease, some other variables are predictive of SpA in children and adolescents; specifically, a family history of SpA, HLA-B27 positivity, and clinically the history or presence of foot enthesopathy and arthropathy uveitis, inflammatory back pain, and sacroiliitis (40–45).

**Figure 1:**
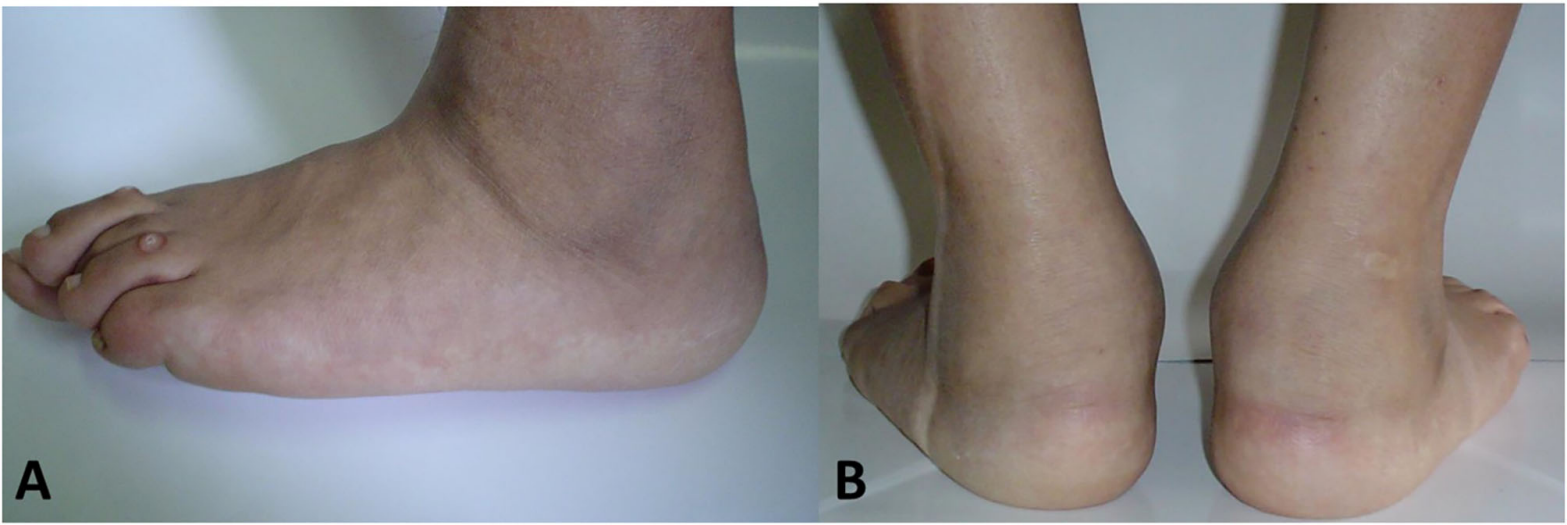
Lateral **(A)** and coronal **(B)** T-2 weighted-fat suppressed MR imaging showing edema in various tarsal bones, joint spaces and soft tissues in a 16 year old boy with chronic ankylosing tarsitis [Modified from Burgos-Vargas (46)].

**Figure 2:**
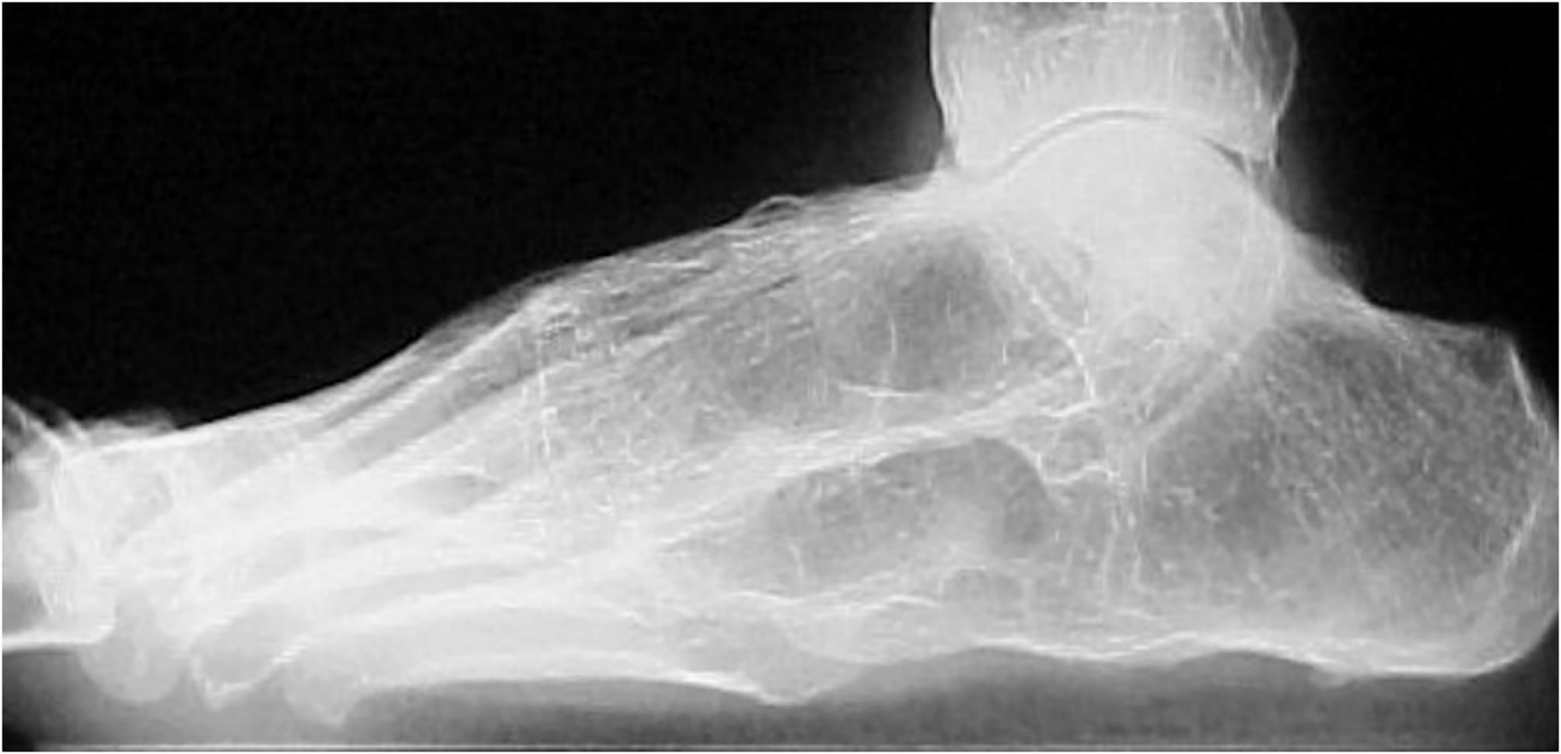
Chronic changes in a patient with JoSpA. Lateral view showing complete tarsal ankylosis and plantar enthesophytosis. Courtesy of Dr. Rubén Burgos-Vargas.

**Figure 3:**
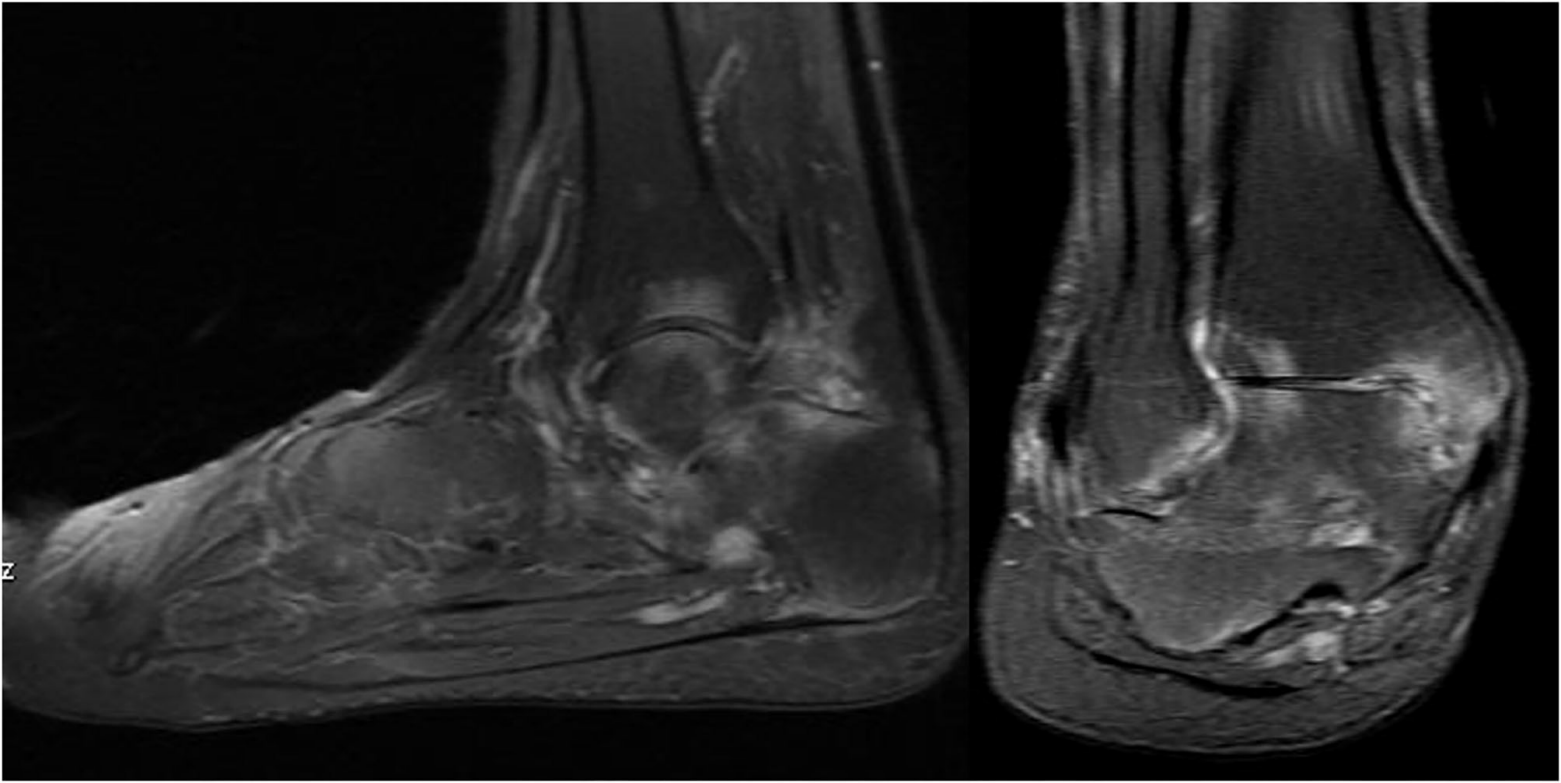
T-2 weighted-fat suppressed MR imaging showing edema in various tarsal bones, joint spaces and soft tissues in a 16 year old boy with chronic ankylosing tarsitis [Modified from Burgos-Vargas (46)].

Tarsitis presents with mid-foot pain and swelling and often swollen ankles, inflammation of the plantar fascia, and Achilles tendon enthesitis (Figure 1). Radiographic studies show a spectrum of findings, such as diffuse osteopenia, joint space narrowing, and bone ankylosis. Erosions and enthesophytes are found in the extraarticular entheses, such as Achilles' tendon and plantar fascia bone attachments (Figure 2). MRI shows bone edema, synovial sheath and bursae swelling, and abundant synovial fluid in the joint space (Figure 3).

The most severe cases are those evolving into ankylosing tarsitis (AT), a condition characterized by a partial or complete fusion of the tarsal bones and by the formation of bone bridges resembling in certain aspects the long-term changes of the sacroiliac and particularly the spine of AS patients (Figure 2).

Besides our descriptions of Mexicans with JoSpA, there are sporadic descriptions of tarsal involvement in other geographic localization and ethnic groups. For example, unilateral ankylosis of the tarsal bones was described in a 19 year-old male with AS diagnosed at the age of 15 who had several mid-foot episodes of arthritis; unilaterality was attributed to radiotherapy (47). In another report, 15 of 40 patients with JoSpA that underwent therapeutic immobilization of the feet developed tarsometatarsal fusion (48).

Chinese and French large cohorts of patients with JoSpA have shown tarsitis in around 6 to 9% (49, 50). Data from India indicate that involvement of the mid-foot is common and severe (17). Likewise, tarsal bone ankylosis was seen in patients with oligoarticular juvenile rheumatoid arthritis and back pain from India (51) and Turkey (52, 53).

Based on the New York classification of sacroiliitis (5, 54), our group has developed an equivalent grading of radiographic parameters of classification and interpretation of tarsitis (39). As a result, the Spondyloarthritis Tarsal Radiographic Index (SpA-TRI) has good sensitivity and specificity to evaluate structural but no inflammatory changes (39, 48).

Tarsal ankylosis (defined as tibiotarsal, intertarsal, or tarsometatarsal ankylosis) occurred in 18% of patients with juvenile rheumatoid arthritis (JRA) and 23% of adult-onset patients with rheumatoid arthritis. In another study, tarsal ankylosis accounted for 25% of the seropositive and seronegative polyarticular JRA, 9 and 19% of the pauciarticular and systemic (51). Interestingly, most of these cases also had carpal ankylosis, and none had SpA. In addition, Tarsal and carpal ankylosis occurred in adult patients with JRA from India (51). Compared with patients without radiographic sacroiliitis, around 40% of such patients with radiographic tarsitis graded 3 or 4 with the SpA tarsal radiographic index (55).

## The Tarsitis Link With SpA

The characteristics of SpA in adults are inflammation and enthesitis, followed by bone proliferation and ankylosis involving the sacroiliac and spinal joints (56). While the cause and mechanisms involved in inflammation are partially known, those participating in bone proliferation remained unidentified. Research advances are associated with new treatment forms, which may induce remission of inflammation but not halter bone formation (57). Additionally, many studies focus on the axial skeleton and very little on peripheral sites. In contrast, striking findings occur in the mid and hindfoot in many animal models, highlighting a window of opportunity to study human samples from peripheral joints.

Unfortunately, pediatric and adult rheumatologists in our clinic and probably from other centers have neglected midfoot involvement despite its severity and consequences. Most people think of the ankle and metatarsophalangeal joints when children and even adults complain of midfoot/tarsus pain and swelling. The connection between the exacerbated inflammatory responses and the abnormal residual ossification remains a potential field to improve our therapeutic approach. Blocking the mechanisms subjacent to bone proliferation could improve the overall prognosis significantly in our patients.

**Figure 4:**
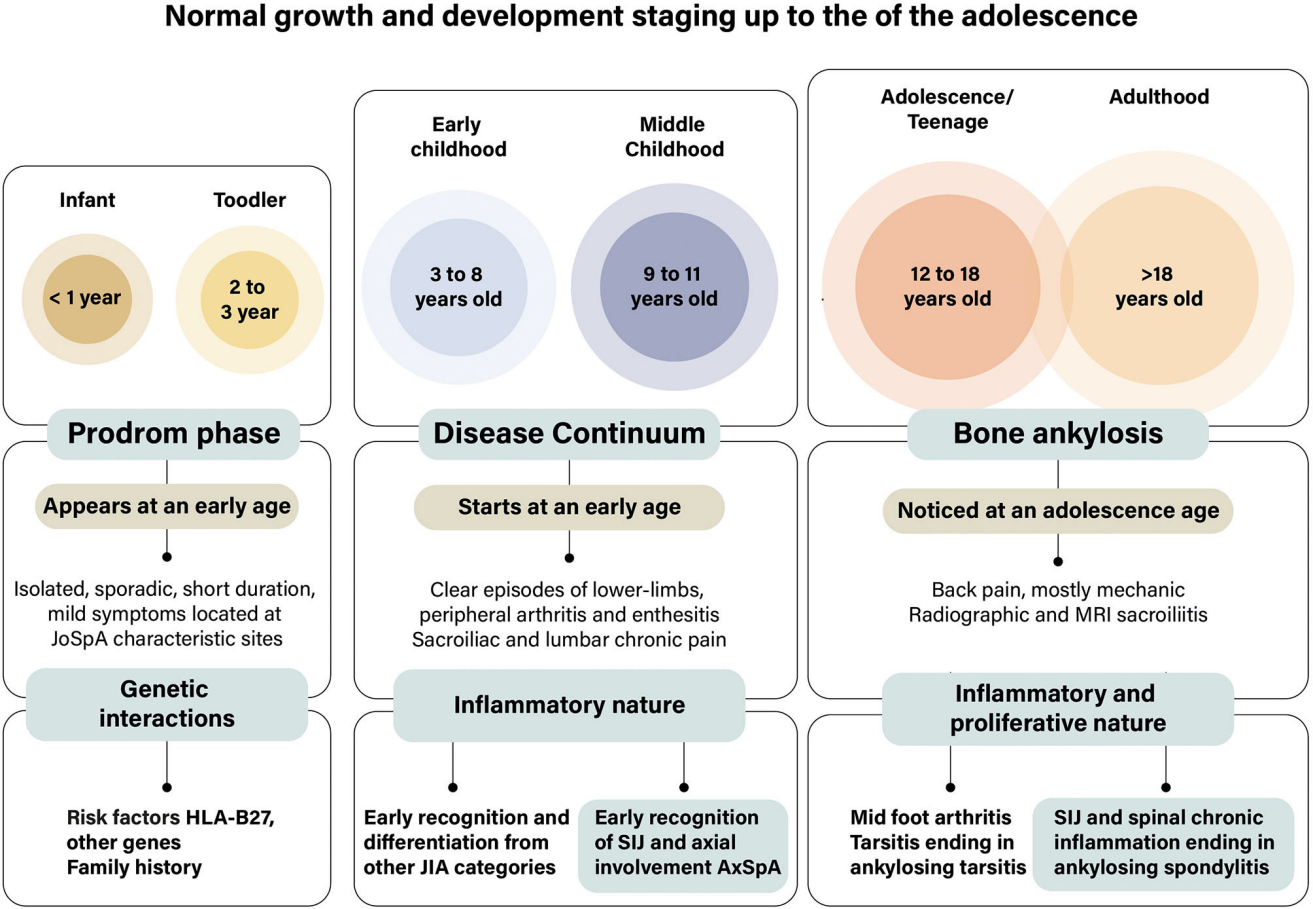
Stages in the development of foot affection in juvenile-onset spondyloarthritis. We proposed three stages to approach the early symptoms of foot affections in patients with JoSpA. The duration of each of the stages is certainly unknown since information is scarce. Few symptoms, such as isolated, sporadic, short duration, episodes of joint pain often considered growing pains, characterize the prodrome stage. Around the age of 8 years starts the inflammatory stage of the disease, which is mostly characterized by arthritis, enthesitis, and axial involvement; this is the disease “continuum” stage, which merged with tarsal ankylosis in the adolescent years or sacroiliac and spinal ankylosis in early adulthood. This is a clinical picture needing differentiation from other diseases, particularly from subgroups of juvenile idiopathic arthritis. At the same time, the relation between children and adult diseases should be established via de sacroiliac and spinal demonstration in children. These events take place when there are several changes in growth and development in childhood and adolescence.

Interest in peripheral arthritis as a critical manifestation of SpA developed in parallel with studies on psoriasis and psoriatic arthritis (PsA) and descriptions of enthesitis and dactylitis (24, 58, 59). Dactylitis often occurred in single toes as a companion to nail psoriasis. Recently, peripheral arthritis appeared again in clinical descriptions (48). An international study of 4,465 patients with SpA found that nearly 70% of the participants had at least one episode of peripheral arthritis (48, 60). Data splitting yielded 57% with arthritis, enthesitis in 44%, and dactylitis in 15%. The study confirmed the highest percentage of peripheral manifestations in around 80% of patients in Latin America, dactylitis in 37% of PsA, and enthesitis in 65% of JoSpA. Mid-foot involvement or tarsitis occurred in rank order in 13% of pSpA, 10% of PsA, 9% of reactive arthritis (ReA) and inflammatory bowel disease (IBD), and 5% of axSpA. The proportion of tarsitis in JoSpA was 19%. Per geographic region, tarsitis occurred in 24% of patients from Latin America. In children, inflammatory clinical events may progress throughout the years and end in bone ankylosis. We proposed three stages to approach the very early symptoms of the “Prodrome” stage, which evolve and progress in a rather slow and recurrent “continuum” of disease, ending up with bone ankylosis (Figure 4).

## Insights Into the Pathogenesis of Ankylosing Tarsitis

There is very scarce information addressing the pathogenesis of peripheral and foot affection on SpA; nonetheless, genetic association studies have revealed that the HLA-B27 predisposition is shared with axSpA and that other genes like LMP2 (61), ERAP-1, ERAP-2 (62) and class II MHC are involved (63).

Although peripheral osteoproliferation seems to be the main problem in these patients, very few studies focus on the pathogenesis of tissue inflammation and proliferation (2). We have previously analyzed tendon, enthesis, and joint samples from the midfoot of Mexican patients with ankylosing tarsitis (AT) (64). Our results revealed a scarce leukocyte infiltration accompanied by an osteoid intrusion in the extracellular matrix (ECM), suggesting that, probably, intramembranous ossification of the enthesis and subchondral osteoproliferation could take place. We also found an important expression of bone lineage proteins like osteopontin (OPN) and osteocalcin (OCN) in mesenchymal tissues as well as parathyroid hormone-related protein (PTHrP) and basic sialoprotein on bone tissues. The role of osteocalcin in physiological and pathological bone formation remains an important question in SpA (65, 66); however, its expression on entheseal cells might involve its participation in inducing an osteoblastic phenotype (67).

## Contribution of Animal Models

Remarkably, in different animal models of SpA, midfoot arthritis and ossification are the main clinical features that can happen either before or simultaneously that axial arthritis. Animal models of transgenic animals like the HLA-B27-transgenic rats develop spontaneous arthritis in the hind paws accompanied by spondylitis, uveitis, and gut inflammation, resembling human disease (68). Interestingly, a transgenic model of TNF overexpression in mice (TNF^Δ*ARE*^) is characterized by Crohn's-like ileitis, midfoot ossification and inflammation, sacroilitis, and spinal ossification (69, 70) that worsens with increased mechanical stress and can develop in the absence of mature T or B cells (71). A more recently described model involves the transmembrane expression of TNF; in this model, animals develop a disease characterized by axial and peripheral enthesitis with abundant leukocyte infiltration (72). These experimental approaches resemble human disease and point to the importance of peripheral arthritis and enthesitis in the onset of the disease with an interesting involvement of immune pathways and mechanical forces.

Another noteworthy model of SpA is induced after the transgenic edition of ZAP70 in SKG (Sakaguchi) mice, which develop SpA and Crohn's-like ileitis after the injection of microbial compounds like curdlan or zymosan (73, 74). In addition, the animals of other induced models like proteoglycan-induced arthritis (PGIA) (75–77), collagen-antibodies induced arthritis (CAIA) (70, 71, 78, 79), and DBA mice (80–85) also can show a certain degree of midfoot inflammation and, in chronic models, a severe ossification, accompanied by overexpression of inflammatory cytokines like IL-1B, IL-12B, IL-17A, and IL-6.

Experimental evidence from the IL-23 minicircle overexpression model points to an essential involvement of tendon and ligaments through altered stromal cell function, myeloid cell responsiveness, or gamma delta (γδ) T cell-dependent mechanisms (78). Furthermore, firm evidence for a link with mechanical stress has arisen from hind limb unloading vs. voluntary running experiments that decreased or increased mechanical stress. The studies firmly showed that both in the TNF^Δ*ARE*^ model (70) and CAIA (71), unloading prohibits arthritis onset, whereas the reverse was observed under voluntary running conditions. Intriguingly, while unloading prohibited the onset of arthritis in collagen-induced arthritis (CIA), it did not interfere with the development of anti-collagen antibodies, indicating that mechanostress regulates joint inflammation but uncouples it from induction of autoantibodies (71). The mechanostress effect is also apparent in the absence of adaptive immunity, suggesting that tissue-resident stroma may account for it. This is intriguing as several studies have pointed to a crucial role for entheseal and skin γδ T cells in the onset of IL-23-driven PsA both on skin and joints (86–88).

Several animal models point that canonical T cells appear not to be indispensable for mechanostress induced inflammation. In line with this, *in vitro* stretch of tendon and ligament-stromal cells induce an array of pro-inflammatory mediators, several of which are shared with skin keratinocytes. They include chemokines, cytokines, and several danger-associated molecular patterns (DAMPs). The induction of CCL2, for example, was shown to recruit classical monocytes. Mechanostress also led to a marked activation of complement, which attenuated mechanostress induced inflammation (89).

**Figure 5:**
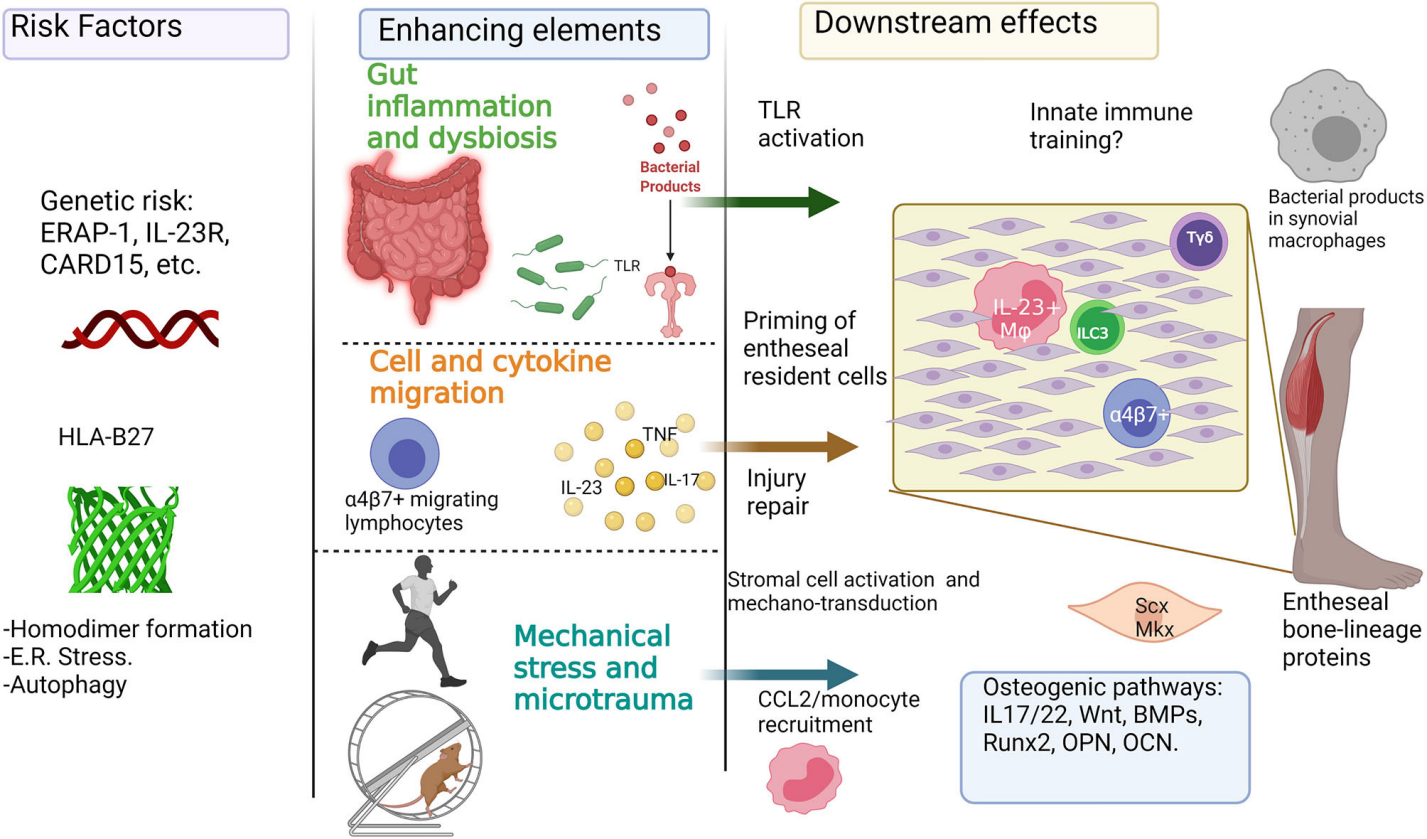
Inflammatory and mechanical factors in the onset of enthesitis: Genetic predisposition for enthesitis can be represented by HLA-B27 and its role in the endoplasmic reticulum (E.R.) stress, autophagy, and homodimer formation, and also by other polymorphisms in genes like ERAP-1 and IL-23R. Further enhancing elements involved in the onset of enthesitis include the role of the “leaky gut” syndrome, intestinal dysbiosis, and the activation of gut-homing lymphocytes. Additionally, mechanical stress can play a key role in enhancing inflammation and osteoproliferation through injury repair mechanisms, production of chemokines like CCL2, and the activation of stromal cells like tenocytes. The previous can be added to the potential role of osteogenic pathways like Wnt, BMP, and Runx2. *This figure was done with biorender (Biorender.com)*.

A summary of the characteristics of animal models that can present midfoot inflammation and ossification is showed in Table 1. Interestingly, many mechanisms can be involved in the development of SpA, and although animal models have provided much of the current information, several studies on human samples reveal a complex immune network that modulates the response to risk factors and enhancing elements toward the onset of the disease (Figure 5).

**Table 1:** Foot and axial involvement in animal models of SpA.

**Experimental model**	**Pathogenic mechanisms**	**Foot involvement**	**Axial involvement**	**Role of mechanical stress**	**Inflammatory pathways**	**Ref**.
Collagen-antibodies induced arthritis (CAIA)	Passive transfer of anti-type II collagen antibodies induces polyarthritis and synovitis	Enthesitis can be found on paws after 7 days of induction	Cartilage hypertrophy, bone damage, and kyphosis can be observed	Mechanical stress drives osteophyte formation and size	Inflammation in this model is dependent on IL-23	(65, 66, 73, 74)
SKG mice	SKG (Sakaguchi) mice have a defect on the SH2 domain of ZAP70 and develop SpA-like disease after the injection of microbial components like Curdlan and Zymosan	Mice develop progressive ankle arthritis and swelling of the foot soft tissue. Foot arthritis and enthesitis precede axial affection	Sacroiliitis, tail, and lumbar arthritis appear after around 10 weeks of induction	Unknown	IL-23 mediates inflammation and bone proliferation. Also, extra-articular manifestations like ileitis and uveitis are involved	(68, 69)
Proteoglycan-induced spondyloarthritis	The injection of bovine proteoglycan and adjuvant induces T cell-induced synovitis with progressive ankylosis	A progressive polyarthritis with synovitis and cartilage destruction is evident in the initial 8 weeks after proteoglycan injection, followed by osteoproliferation and ankylosis of frontal and hind limbs	Spinal arthritis starts weeks after peripheral arthritis and ankylosis, followed by intervertebral ankylosis and bone proliferation	Unknown	T-cell induction of arthritis and TNF involvement	(70–72)
HLA-B27 transgenic rats	Transgenic expression of human HLA-B27 and β2-microglobulin in rats induces spontaneous arthritis, spondylitis, ankylosis, and gut inflammation	Tarsal affection is classically described as tenderness, swelling, and inflammation of one or two hind limbs	Axial bone proliferation and sacroiliitis are present	Unknown	Several mechanisms have been described, including HLA-B27 misfolding, homodimer formation, gut dysbiosis, and type 3 immunity pathways	(63)
TNF^Δ*ARE*^ Mice	A deletion in the regulators of TNF (AU-rich elements, or ARE) induces a prolonged overexpression of TNF, inducing gut inflammation, and a SpA-like phenotype	Arthritis initiates at enthesis in interphalangeal joints and Achilles' tendon	Radiographic spinal inflammation and sacroiliitis are evident after 4 months of development	Tail unloading experiments demonstrated that mechanical stress is an essential driver of arthritis	The model can be induced in the absence of mature T and B cells so that stromal cells might drive the mechanisms leading to arthritis and enthesitis	(64–66)
Transmembrane TNF	A defect in the cleavage site for ADAM17 drives overexpression of membrane-bound but not soluble TNF	Peripheral enthesitis and osteitis are accompanied by bone proliferation with entheseal and synovial leukocyte infiltration	Animals develop tail deformities and spondylitis with deformation with focal joint destruction. Also, inflammation of the spinal ligaments and bone marrow is present	Unknown	The inflammation is driven by TNF-receptor I and can be induced with the TNF overexpression of only stromal cells	(67)
Spontaneous arthritis in DBA Mice	Hormonal, aging, and behavioral factors are involved in the spontaneous development of arthritis, enthesitis, and ankylosis of 4 month old DBA/1 male mice	Initial signs are detected in interphalangeal, metatarsophalangeal, and ankle joints with further swelling of tarsal joints. Enthesitis, dactylitis, and psoriasiform nail changes are frequently observed	Scarce evidence of axial ankylosing or enthesitis	An aggressive behavior related to mice fight and microtrauma is involved; nevertheless, the specific role of the mechanical load has not been explored	There is evidence of a role of testosterone, BMP signaling, and inflammatory cytokines like IL-1, IL-12, IL-6, and IL-17. Arthritis and enthesitis can be induced in the absence of alpha-beta or gamma-delta T cells	(75–80)

## Inflammatory Pathways in Axial and Peripheral SpA

Genetic studies have provided a significant step in the discovery of potential triggers of SpA pathogenesis. The most studied gene for SpA susceptibility is the class I histocompatibility molecule HLA-B27 (90, 91), which, previously was considered as a potential link with “arthritogenic peptides” presented to self-reactive lymphocytes; nevertheless, this has not been proven, and current theories postulate other roles like the induction of endoplasmic- reticulum (ER) stress (92) and homodimer formation (93).

Studies in animal models of transgenic HLA-B27 rats and mice (68) revealed that this molecule tends to misfold during its synthesis in the endoplasmic reticulum (ER) (94), causing ER stress, activation of the unfolded protein response (UPR) (95) and the induction of the inflammatory cytokine IL-23 (92). However, UPR activation has not been proven in humans, as UPR markers are not increased in samples of patients of SpA (96), and instead, some reports suggest that IL-23 production could be related to an increase of autophagy markers in the gut (97). Furthermore, killer immunoglobulin receptors (KIR) expressed on NK and Th17 cells can recognize aberrant B27 homodimers (98, 99), and therefore, induce IL-17 production. Remarkably, heavy chain homodimers have been found in patients' gut and vertebral joints (100).

Although most of the cellular and molecular mechanisms related to the initial triggers and the amplification of inflammation in SpA are known for animal models and *in vitro* research, the use of bDMARDs has allowed basic and clinical researchers to study the role of several cytokines in the human SpA. The first and most known biologic target is the pro-inflammatory cytokine TNF, and TNF inhibitors (TNFi) are currently the most used treatment for the disease. The use of TNFi confirmed its therapeutic effect in adult patients with AS (38) and prevented proliferation and structural progression after 8 years (101). Although JoSpA patients can also benefit from TNFi (102), there are no reports of osteoproliferation in these patients' peripheral joints.

Type 3 immunity (mediated by IL-23, IL-17, and IL-22) also plays an essential role in the pathogenesis of SpA. IL-17 is a pro-inflammatory cytokine involved in animal models of SpA (74, 78, 99) and increased in patients' blood and synovial fluid (103–106). The pro-inflammatory and destructive effects of IL-17 have been associated with synovitis, enthesitis, and bowel inflammation (107–109). Also, animal models of IL-23 minicircle injection (78) and SKG mice (74) mainly depend on IL-17. The therapeutic inhibition of this cytokine has significant effects on disease activity and vertebral inflammation (110), although, again, few reports focus on peripheral symptoms, and the existing ones only evaluate PsA patients (111, 112).

IL-23 is a cytokine involved in the differentiation and maintenance of the Th17 phenotype (113). Its role in SpA has become highly relevant for the scientific community since, in 2012, it was published that the over-expression of this cytokine with DNA minicircles in mice could induce a spontaneous model of SpA-like disease (78). In the report by Sherlock *et al*., this cytokine could act on a previously undescribed population of entheseal resident CD3+, RORγt+, Sca1+, CD4–, CD8–, IL−23R+ T cells. Several groups around the world have tried to identify such entheseal, IL-23 responsive cells, and it has been suggested that invariant-receptor natural-killer T cells (iNKT) (114), Th17 (99), mucosal-associated invariant T (MAIT) cells (107), Tγδ (103), and type 3 innate lymphoid (ILC3) cells (104) could be responsible for the IL-23 role on inflammation and osteoproliferation. Strikingly, when the inhibition of IL-23 was taken to the clinics, it did not result in any therapeutic benefit (115), suggesting that probably, there are IL-17 producing cells independently of the IL-23 status (116, 117) or that IL-23 is involved in very early steps of SpA induction (118).

Almost half of the patients with SpA have microscopical subclinical gut inflammation (119), and there is a strong association of IBD with SpA. Whether or not this relationship is involved in the foot involvement of JoSpA remains unknown. Nonetheless, the increased severity and frequency of tarsitis in developed countries is probably associated with a higher incidence of intestinal infections. The relationship between gut inflammation and arthritis or enthesitis is a challenging research topic that includes intestinal dysbiosis and cell migration (120–122). It has been suggested that some inflammatory mediators like cytokines and leukocytes can be originated in the gut, and such could be the case of ILC3 cells (104) or Tγδ cells (123).

Recently, our group described that a population of Tγδ cells expressing the gut-homing integrin a4β7 is enriched in the peripheral blood of patients with axSpA and that this population has an increased expression of TLR2 and TLR4, which might induce them to a pre-activation state and an enhanced response to pathogenic molecular patterns (123). The characteristics of this population are still unknown; nevertheless, further studies are needed to address a possible migration phenomenon.

SpA has been linked to a strong genetic predisposition (124, 125) and certain micro-organisms interplay with the immune system (126, 127), with reactive arthritis as a prototypic example, although this association is not always that clear in a significant fraction of patients. Similarly, what drives the joint-centered inflammation in this spectrum of diseases has been a longstanding enigma in the field. Previous work from our group has revealed an interplay between infections and SpA (128). Specifically, we described the presence of bacterial DNA in synovial macrophages (129) and antibody and cellular immune response against the enterobacterial heat shock protein-60 (HSP60) in blood and synovial fluid samples (127).

While many residual questions remain to date, some recent concepts have arisen that at least partially address why some joints or joint structures is typically associated with spondyloarthritis. Spondyloarthritides is notoriously known to affect enthesis, particularly those of lower limbs such as Achilles Tendons or *fascia plantaris* (130, 131). This is a feature that at least clinically is considered a hallmark of the spondyloarthritides spectrum. These clinical concepts are supported by a large body of imaging data demonstrating not only soft tissue swelling but also associated osteitis. These observations highlight the importance of the functional unit formed between muscle, tendons, the entheseal part, and the underlying bone. The tissues connecting muscle to bone (tendons) or bone to bone (ligaments) are specialized to permit the transmission of mechanical forces. Despite this, few mechanistic studies have addressed how mechanical forces may drive the onset of joint inflammation.

## Mechanical Forces Might Drive Entheseal and Articular Inflammation

Healthy tendons and ligaments contain several unique cell types to ensure their homeostasis. They contain stromal cells, referred to as tenocytes, that constitute the majority of cell types within healthy tendons and ligaments. Their primary role is to control the extracellular matrix synthesis by producing collagen or degrade them by releasing proteases (132, 133). Tenocytes are notoriously mechanosensitive cells mediated at least in part by the transcription factors scleraxis (Scx) and mohawk (Mkx), which drive the expression of mechanical stress-activated genes, extracellular matrix genes (e.g., collagen) or adhesion molecules. These elements can interact with the circulating or resident immune system, and although enthesis resident immune cells are scarce, there are rare T cell subsets such as γδT cells and ILC3s, also, IL-23 producing CD14+ myeloid cells have been described (133).

The role of these immune cells in a steady state is relatively poorly understood but is thought to play a role in tendon and ligament repair. Despite the undeniable role of mechanical loading on tendon and ligament homeostasis in health, several observations have indicated that mechanical stress also leads to inflammation. Thus, healthy individuals exposed to intense physical activities (athletes, military recruits) may often develop bone marrow edema on sacroiliac joint imaging with many resemblances to acute inflammatory lesions noted in SpA patients (134, 135). Not surprisingly, mechanical stress has also been linked to inflammatory rheumatic diseases such as RA, PsA, and AS (136). Here, physical trauma has been associated with disease initiation and structural progression (137, 138).

In sum, mechanical forces display a myriad of effects on skin, tendon, and ligaments, reflecting a crosstalk between stromal and immune cells. However, there are still several outstanding questions. The threshold between normal mechanical loading and pathological stress is poorly defined, and whether mechanostress-induced inflammation in PsA reflects altered response to normal or rather exposure to supraphysiological levels of mechanical stress is currently unclear (139). Alternatively, the resolution of inflammation induced by mechanostress may also be impaired, although the underlying mechanisms are still relatively unclear.

Anatomically, the foot has 28 bones and 31 joints into three large areas: the forefoot (metatarsals and phalanges), the mid-foot (cuboid, navicular, and cuneiforms), and the hindfoot (calcaneus and talus). The foot and the pelvis are the most important weight-bearing structures in spreading loads through the spine, lower limbs, the tarsal areas, and the foot arches. Therefore, mechanical forces could drive structural damage in a similar way to the experimental models described before.

Certainly, future studies are pointing to a potential role of mechanical stress and microtrauma on inflammation and bone formation; However, some questions remain open about the interaction of these elements in the initiation of tissue repair mechanisms and ossification, there is a need to explore if inflammation and mechanostress act as sequential factors, enhancing elements or independent pathways.

## Bone Formation in SpA

Although little is known regarding the specific mechanisms of osteoproliferation in SpA, this phenomenon is probably derived from inflammation, according to radiographic studies. It has been postulated that HLA-B27 homodimers can be recognized by killer immunoglobulin-like receptors (KIR) expressed on Th17 and NK cells and that these cells can produce IL-17 mediated responses that link HLA-B27 with inflammation (91, 140). These homodimers have been found in spinal joints; nevertheless, it has not been explored if they can be found in peripheral joints.

The cytokine IL-22 is a master regulator of epidermal proliferation and barrier integrity, both in the gut and the skin; this capacity to induce proliferation is not restricted to epidermal tissues, as it has shown to interact with joint stromal cells. In the mice model of IL-23 overexpression (78), it has been reported that IL-22 (which can be either produced downstream of the IL-23 effect or independently) can induce the expression of bone growth molecules such as Akp3, Cebpb, Wnt10b, Wnt3a, and Gli1. This cytokine can also induce the mineralization and ossification of mesenchymal stem cells (141) and induce keratinocyte and fibroblast proliferation in psoriasis (142). IL-22 can become a potential therapeutic target to prevent the bone formation in the spondyloarthritides, although its effect on barrier integrity and host defense make this a difficult step.

Remarkably, there are two pathways of bone formation, endochondral and pseudomembranous ossification, and both can participate in the pathogenic bone formation of SpA patients (67, 143–145). Intense research has elucidated some pathways related to bone formation in SpA, such as the Wnt and bone morphogenetic protein (BMP) pathways (84, 146, 147). Results from the animal model of aging DBA/1 mice showed that the injection of the BMP-inhibitor nogging significantly decreases ossification and entheseal cell formation at peripheral joints. The role of BMP in peripheral SpA is also reinforced by the evidence of smad 1/5 activity in Achilles tendon's enthesis samples of SpA patients (84).

The Wnt signaling pathway is a key regulator of bone formation that can be altered in diseases like osteoarthritis (OA), rheumatoid arthritis (RA), and SpA (146). Remarkably, the Wnt inhibitor Dickkopf-1 (Dkk-1) is downregulated in patients with AS (148); therefore, a higher Wnt activity has been related to a pro-osteogenic phenotype as reviewed elsewhere (146, 149).

Sclerostin (encoded by the *sost* gene) is another Wnt inhibitor that can also inhibit BMP function (150) with an important role in SpA pathogenesis. Immunohistochemical analysis of zygapophyseal joints of SpA patients has revealed a very low sclerostin expression compared to samples from OA and RA patients or healthy subjects (151). The levels of sclerostin and anti-sclerostin antibodies can be useful as biomarkers for axial disease (152). However, there are no reports of its involvement in peripheral affection and tarsal ankylosis.

Although the question about the effect of inflammation and biologic therapy in bone formation is a controversial topic (153), preclinical studies revealed that TNF inhibitors could modulate Dkk-1 activity (148) and strikingly, recent studies suggest that the inhibition of the IL-12 and IL-23 pathway with Ustekinumab can increase Wnt activity (154).

The potential role of inflammation and mechanical stress in the onset of peripheral enthesitis is shown in Figure 5.

## Concluding Remarks and Future Directions

Even though peripheral symptoms of SpA and, particularly those of juvenile-onset patients, are widely recognized, there are still many questions regarding the behavior of structural evolution, bDMARD response, association with gut dysbiosis/microbiota, and immune-mediated pathogenesis. There is a current need for more profound studies in all these fields, considering that demographic and clinical characteristics might be recognized and considered as core manifestations of the disease. In this review, we emphasized the critical role of foot affection in JoSpA patients. We attempted to focus on these manifestations as potential early diagnostic elements and on these manifestations as potential early diagnostic elements and prospective sites for translational studies.

The peripheral approach of areas in which physiopathological events occur warrants a potential site for *in-situ* study of the SpA, providing a remarkable opportunity to deepen on the mechanical triggers that influence the proliferation of what can be considered as a dynamic anatomic-functional “foot unit.” The relationship between gut dysbiosis and peripheral SpA is still poorly understood. Although many reports analyze these factors separately, there is a lack of integrating elements that explain this interaction. Moreover, the effect of mechanical stress probably acts as an enhancing factor for previously primed immune and environmental elements.

## Author Contributions

RB-V conceived the idea and directed the project. JR-L and RB-V revised the final version and prepared the figures. All the authors contributed equally to the writing of the manuscript.

## Funding

JR-L, CP-T, and RB-V receive a scholarship from Sistema Nacional de Investigadores (SNI- CONACYT). JR-L receives support from PEE, PEI, and SIJA-UNAM.

## Conflict of Interest

The authors declare that the research was conducted in the absence of any commercial or financial relationships that could be construed as a potential conflict of interest.

## Publisher's Note

All claims expressed in this article are solely those of the authors and do not necessarily represent those of their affiliated organizations, or those of the publisher, the editors and the reviewers. Any product that may be evaluated in this article, or claim that may be made by its manufacturer, is not guaranteed or endorsed by the publisher.
